# Ubiquitination Is Required for Effective Replication of Coxsackievirus B3

**DOI:** 10.1371/journal.pone.0002585

**Published:** 2008-07-09

**Authors:** Xiaoning Si, Guang Gao, Jerry Wong, Yahong Wang, Jingchun Zhang, Honglin Luo

**Affiliations:** 1 The James Hogg iCAPTURE Centre for Cardiovascular and Pulmonary Research, Department of Pathology and Laboratory Medicine, University of British Columbia, Providence Heart + Lung Institute, St. Paul's Hospital, Vancouver, British Columbia, Canada; 2 Chinese Internal Medicine Laboratory, Department of Cardiology, Dongzhimen Hospital Affiliated with Beijing University of Chinese Medicine, Beijing, China; University of Hong Kong, China

## Abstract

**Background:**

Protein ubiquitination and/or degradation by the ubiquitin/proteasome system (UPS) have been recognized as critical mechanisms in the regulation of numerous essential cellular functions. The importance of the UPS in viral pathogenesis has become increasingly apparent. Using murine cardiomyocytes, we have previously demonstrated that the UPS plays a key role in the replication of coxsackievirus B3 (CVB3), an important human pathogen associated with various diseases. To further elucidate the underlying mechanisms, we examined the interplay between the UPS and CVB3, focusing on the role of ubiquitination in viral lifecycle.

**Methodology/Principal Findings:**

As assessed by *in situ* hybridization, Western blot, and plaque assay, we showed that proteasome inhibition decreased CVB3 RNA replication, protein synthesis, and viral titers in HeLa cells. There were no apparent changes in 20S proteasome activities following CVB3 infection. However, we found viral infection led to an accumulation of protein-ubiquitin conjugates, accompanied by a decreased protein expression of free ubiquitin, implicating an important role of ubiquitination in the UPS-mediated viral replication. Using small-interfering RNA, we demonstrated that gene-silencing of ubiquitin significantly reduced viral titers, possibly through downregulation of protein ubiquitination and subsequent alteration of protein function and/or degradation. Inhibition of deubiquitinating enzymes apparently enhances the inhibitory effects of proteasome inhibitors on CVB3 replication. Finally, by immunoprecipitation, we showed that coxsackieviral polymerase 3D was post-translationally modified by ubiquitination and such modification might be a prerequisite for its function in transcriptional regulation of viral genome.

**Conclusion:**

Coxsackievirus infection promotes protein ubiquitination, contributing to effective viral replication, probably through ubiquitin modification of viral polymerase.

## Introduction

Coxsackievirus B3 (CVB3), a small RNA virus in the *picornaviridae* family, is an important human pathogen associated with various diseases, including myocarditis, aseptic meningitis, pancreatitis and possibly insulin-dependent diabetes. We and others have shown that CVB3 infection leads to activation of several intracellular signaling pathways [1]–[7], and downregulation of host proteins likely through the ubiquitin/proteasome system (UPS) [7]–[9].

It is well-established that the UPS is the major intracellular proteolytic system of all eukaryotic cells [10], [11]. The ATP-dependent system begins with covalent attachment of ubiquitin to the ubiquitin-activating enzyme (E1). Then the ubiquitin is transferred to a ubiquitin-conjugating enzyme (E2). Finally, ubiquitin ligase (E3) transfers the ubiquitin to the substrate protein. After several cycles of ubiquitination, multiple ubiquitin molecules are attached to the substrate which is then quickly recognized and subsequently degraded by the 26S proteasome. Ubiquitin is recycled through the actions of deubiquitinating enzymes (DUBs) [12], [13]. There are at least two classes of deubiquitinating enzymes, the ubiquitin C-terminal hydrolases (UCHs) and ubiquitin-specific processing proteases family.

In addition to the degradation of mutant, damaged and misfolded proteins, this system is responsible for the modulation of many regulatory proteins such as cyclins [14], inhibitors of cyclin-dependent kinases (p21, p27) [15], tumor suppressors (p53) [16], and inhibitor of NFκB (IκB) [17], which are essential for a variety of cellular functions, including cell-cycle regulation, apoptosis and host immune responses [18]. Unlike polyubiquitination in the regulation of protein degradation, monoubiquitination of cellular proteins, such as histones, calmodulins, actin, proliferating cell nuclear antigen and receptor tyrosine kinases, plays more diversified roles involving in the regulation of chromatin remodeling, DNA repair, transcriptional regulation and endocytosis [19].

Since the first discovery that human papillomavirus protein E6 targets the cellular tumor suppressor protein p53 for the UPS-mediated degradation [16], increasing studies, including those from our laboratory, have suggested that various viruses evolve different mechanisms to utilize or manipulate the host UPS for their own benefits [9], [20]–[25]. We have previously shown that CVB3 infection results in downregulation of several host proteins [7], [9], such as cell-cycle protein cyclin D1, tumor suppressor p53, and transcription activator β-catenin in infected HeLa cells. The downregulation of host proteins following CVB3 infection is most likely through the UPS. Specific inhibitors to 26S proteasome reverse the degradation of proteins in HeLa cells [7], [9] and reduce CVB3 replication in murine cardiomyocytes [26].

In this study, we investigated the possible underlying mechanisms by which the UPS regulates CVB3 replication. We demonstrated that protein ubiquitination was enhanced after coxsackievirus infection. We further showed that knockdown of ubiquitin expression by small-interfering RNA (siRNA) decreased CVB3 infection, likely through the downregulation of ubiquitination and subsequent alteration of protein function and/or degradation. In addition, we showed that inhibition of deubiquitinating enzyme increased the inhibitory effects of proteasome inhibitors on CVB3 replication. We also found that CVB3 RNA-dependent RNA polymerase 3D (3D^pol^) was modified by ubiquitination. Taken together, our study suggests an important role of ubiquitination in the regulation of coxsackieviral replication.

## Results

### Proteasome inhibition reduces CVB3 infection in HeLa cells

**Figure 1:**
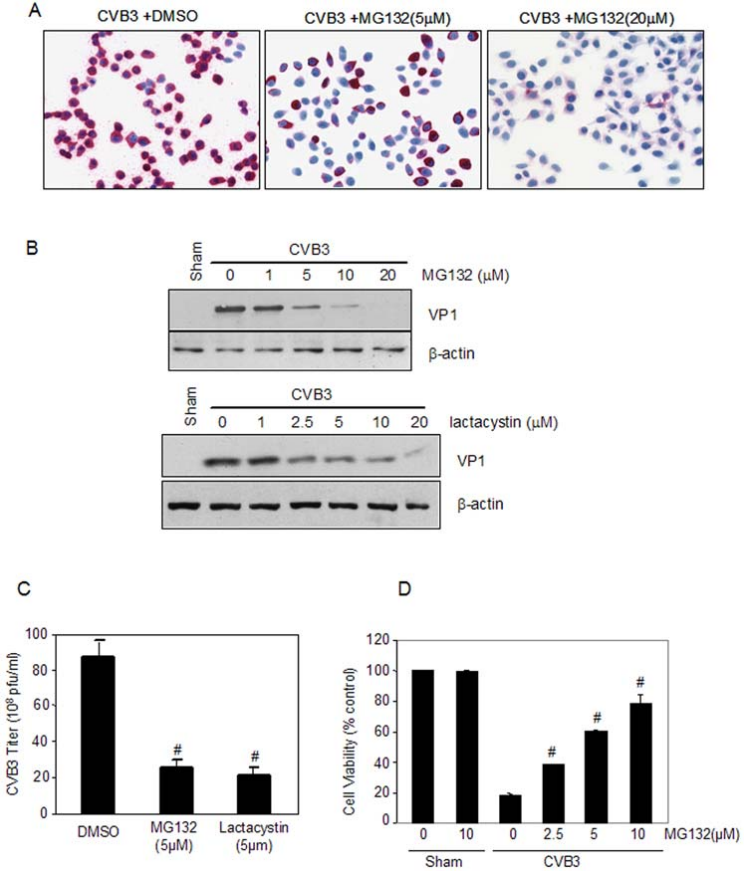
Proteasome inhibitors decrease coxsackieviral RNA expression, viral protein synthesis and viral progeny release in HeLa cells. HeLa cells were sham-infected with PBS or infected with CVB3 in the presence or absence of MG132 or lactacystin. (A). Seven hours post-infection (pi), positive-stranded viral RNA was determined by *in situ* hybridization using anti-sense riboprobes for CVB3 (red). Cell nuclei were counterstained with hematoxylin (blue). (B). Cell lysates were collected at 7 h pi and immunoblotted with anti-VP1 and anti-β-actin (loading control) antibodies. (C). Medium was collected from CVB3-infected cells at 16 h pi and virus titer was determined by plaque assays. The data shown are mean±SE (standard errors) from three independent experiments. ^#^ p<0.001 as compared to CVB3 infection without treatment. (D). Cell viability assay was performed at 16 h pi by the MTS assay which measures mitochondrial function (mean±SE, n = 3). One hundred percent survival was defined as the level of MTS in sham-infected cells in the absence of MG132. ^#^ p<0.001 as compared to CVB3 infection only without MG132 treatment.

To uncover the underlying mechanisms of the antiviral activities of proteasome inhibitors, we chose to use the well-characterized HeLa cells to further our study. We first examined the role of proteasome inhibition in CVB3 replication. As shown in Fig. 1, we found that proteasome inhibitor, MG132, significantly reduced CVB3 viral RNA synthesis (Fig. 1A). Both proteasome inhibitors used in the study, MG132 and lactacystin, decreased the synthesis of CVB3 capsid protein, VP1, in a dose-dependent manner (Fig. 1B). In addition, two inhibitors inhibited CVB3 viral titers by up to fifteen folds (Fig. 1C). Although MG132 and lactacystin significantly inhibited cellular 20S proteasome activities, we have previously demonstrated there was no apparent difference in proteasome activities between CVB3-infected and sham-infected HeLa cells [9]. Together, these results suggest that efficient replication of CVB3 requires the intact UPS function rather than the core proteasome activity alone.

We also performed cell viability assay and morphological examination to determine whether inhibiting viral replication by proteasome inhibitors is due to the toxicity. We found that there was no measurable cell death throughout the incubation period for all doses of proteasome inhibitors used in this study (Fig. 1D). On the contrary, virus-induced cell death was markedly inhibited after the treatment of proteasome inhibitors as a result of decreased viral replication (Fig. 1D).

### CVB3 infection promotes protein ubiquitination

**Figure 2:**
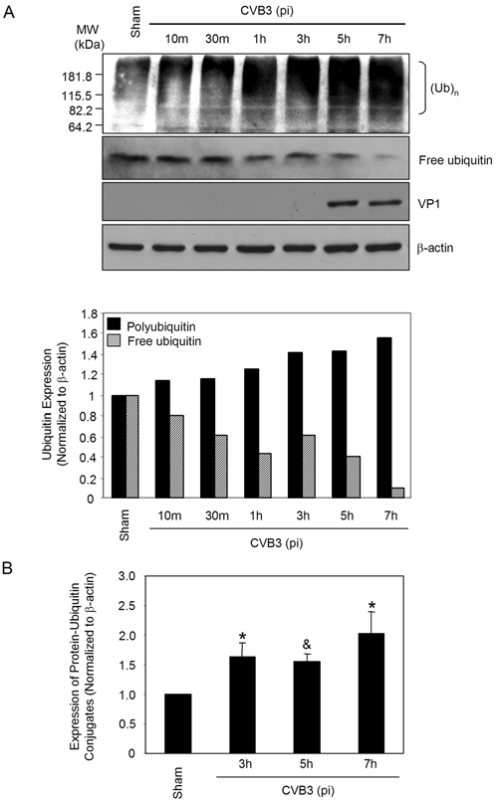
CVB3 infection results in increased protein polyubiquitination and decreased free ubiquitin. (A). HeLa cells were infected with CVB3 or sham-infected with PBS. At different timepoints after viral infection, cell lysates were collected and immunoblotted with anti-ubiquitin, anti-VP1 and anti-β-actin (loading control) antibodies as described in “Material and Methods”. Protein levels of protein-ubiquitin conjugates (molecular weight starting from 82.2 kDa to approximately 230 kDa) and free ubiquitin were quantitated by densitometric analysis using NIH ImageJ program and normalized to the sham infection, which was arbitrarily set to a value of 1.0. Similar results were observed in two independent experiments. (B). Statistical analysis of protein-ubiquitin conjugates at 3 h, 5 h and 7 h after CVB3 infection. The data represent mean±SE of five different experiments. * p<0.05; ^&^ p<0.01 as compared to protein expression in sham infection.

As alluded to earlier, two successive steps are involved in protein degradation: (1) covalent attachment of ubiquitins to the target protein substrate, and (2) degradation of the polyubiquitinated protein by the 26S proteasome with the release of ubiquitin for recycling. To dissect out the role of ubiquitination and degradation in CVB3 infection, we next decided to investigate the protein ubiquitination after CVB3 infection. As shown in Fig. 2A, protein ubiquitination was gradually increased along the time-course of CVB3 infection, which was accompanied by a decrease of free ubiquitin levels. Densitometric analysis further demonstrated that the increases in protein ubiquitination at 3 h, 5 h, and 7 h post-infection were statistically significant as compared to sham infection (Fig. 2B). We have previously demonstrated that 26S proteasome activities were unchanged during CVB3 infection [9]. Thus, the finding of increased accumulation of ubiquitinated proteins is likely due to enhanced protein ubiquitination as opposed to reduced proteasome activity. Decreased levels of free ubiquitin could be a direct consequence of the increased protein ubiquitination. These results suggest that enhanced ubiquitin conjugation may be a prerequisite for efficient synthesis of CVB3 viral RNA and continuation of its lifecycle.

### Knockdown of ubiquitin by siRNA reduces CVB3 infection

**Figure 3:**
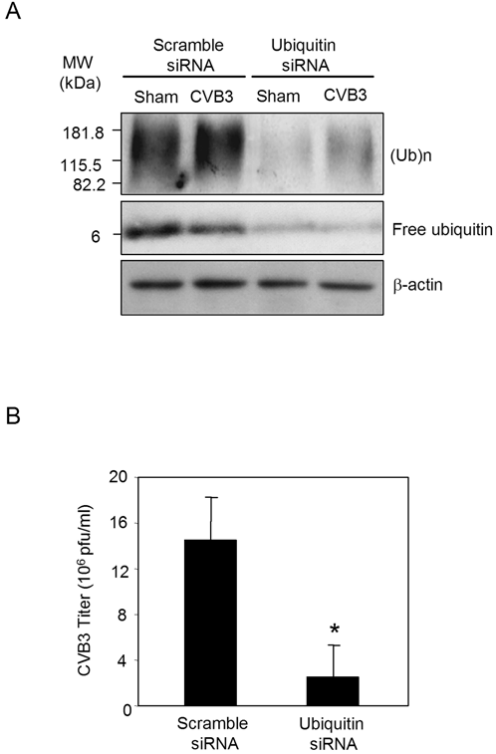
Knockdown of ubiquitin expression by siRNA reduces CVB3 replication. HeLa cells were transiently transfected with the ubiquitin siRNA or a scramble control siRNA. Twenty-four hours post-transfection, HeLa cells were infected with CVB3 or sham-infected with PBS. Cell lysates were collected at the indicated timepoints. (A). Immunoblot was performed with anti-ubiquitin and anti-β-actin (loading control) antibodies. (B). Supernatants of infected cells were collected at 7 h pi to measure CVB3 progeny virion release by plaque assay (Mean±SE, n = 4). Results represent data from three independent experiments. * p<0.05 as compared to virus titers in scramble siRNA-transfected cells.

In addition to blocking proteasome proteolytic activities, proteasome inhibitors are known to reduce free ubiquitin levels in treated cells [27]. It has been suggested that proteasome inhibition negatively affects the budding of retroviruses through reducing free ubiquitin level and subsequently interfering with ubiquitination of viral Gag proteins [22], [24], [25]. Ubiquitin is generated in the cell by proteolysis of polyubiquitinated proteins or ubiquitin fused to carboxyl extension proteins (CEPs) [28]. To investigate whether protein ubiquitination is beneficial to CVB3 replication in HeLa cells, we used the ubiquitin-specific siRNA to gene-silence the expression of human ubiquitin-CEP Uba80, which codes for ubiquitin fused to ribosomal protein S27a [29]. As shown in Fig. 3A, both ubiquitin conjugates and free ubiquitin levels were markedly knocked down after the treatment of ubiquitin siRNA. We further showed that viral titers were significantly reduced in the ubiquitin siRNA-transfected cells as compared to scramble siRNA control (Fig. 3B), suggesting that protein ubiquitination is a critical process adopted by coxsackievirus for the successful completion of its lifecycle.

### DUB inhibition further enhances the inhibitory effects of proteasome inhibitor on CVB3 replication

**Figure 4:**
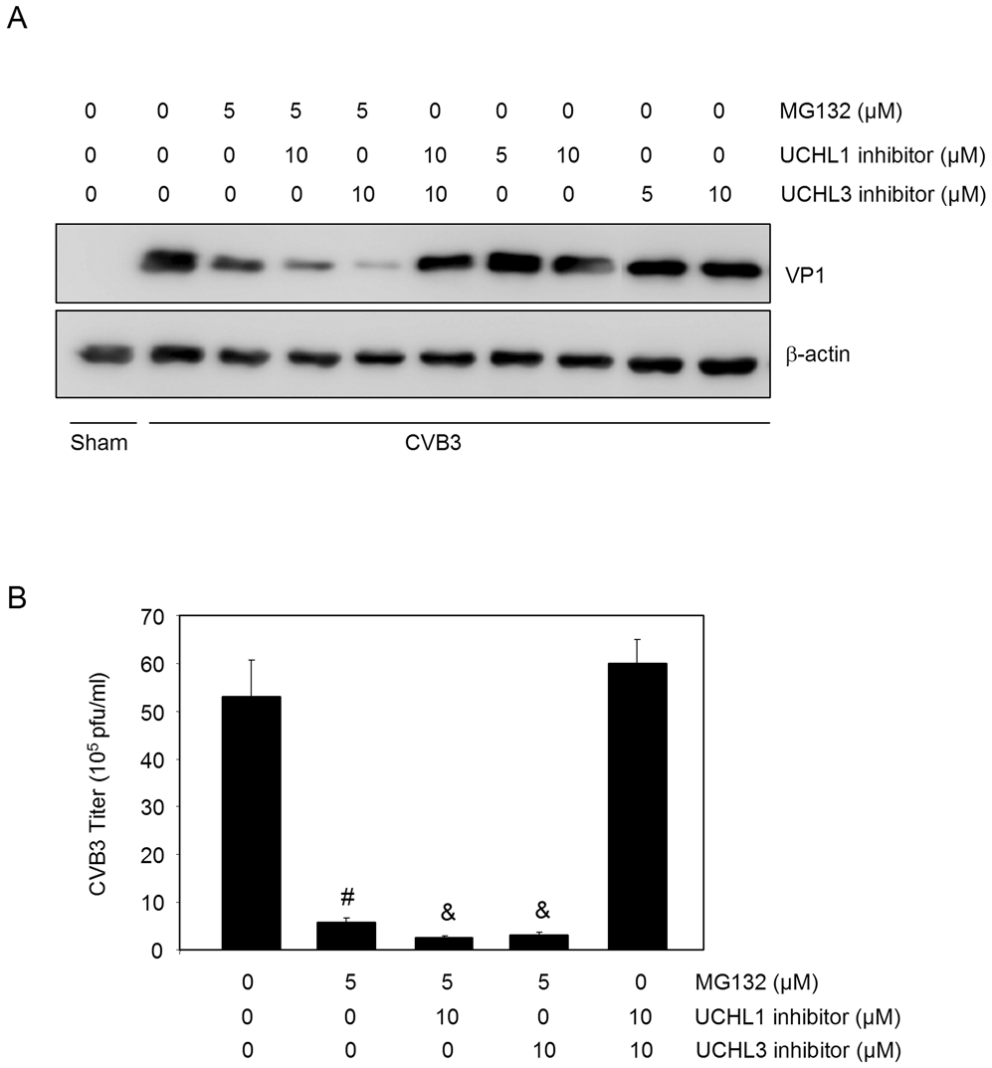
DUB inhibition further enhances the inhibitory effect of proteasome inhibitors on CVB3 replication. HeLa cells were infected with CVB3 or sham-infected with PBS, UCH L1 inhibitor, UCH L3 inhibitor and proteasome inhibitor MG132 were added 1 h pi as indicated. Seven hours pi, cell lysates and supernatant were collected for immunoblotting and plaque assay, respectively. (A). Immunoblot was performed using anti-VP1 and anti-β-actin (loading control) antibodies. Similar results were observed in two independent experiments. (B). Virus titer was measured by plaque assay (Mean±SE, n = 4). Results represent data from three independent experiments. ^#^ p<0.001 as compared to CVB3 infection only without treatment; ^&^ p<0.01 as compared to MG132 treatment alone.

It has been demonstrated that protein ubiquitination can also be regulated by deubiquitinating enzymes that specifically cleave ubiquitin from ubiquitin-conjugated protein substrates [12], [13], [30]. To further explore the role of protein ubiquitination in viral replication, we examined the influence of DUB inhibition on viral protein expression. Two commercially available ubiquitin c-terminal hydrolase inhibitors, UCH L1 and UCH L3 inhibitors, were used for this study. As shown in Fig. 4, specific inhibition of UCH L1 or UCH L3 further reduced CVB3 protein expression and virus titers in proteasome inhibitor-treated cells, suggesting that these enzymes may be involved in the lifecycle of CVB3. Nevertheless, it was found that inhibition of the UCH L1 and L3 activities alone was not sufficient to block coxsackievirus replication since no significant changes in viral protein expression and CVB3 titers were observed in cells treated with two UCH inhibitors either separately or in combination (Fig. 4). As discussed earlier, stabilization of short-lived host proteins and prevention of protein ubiquitination by reducing recycled ubiquitin likely contribute to the inhibitory effect of proteasome inhibition on viral replication. Thus, it is speculated that DUB inhibition by UCHL1/L3 inhibitors alone, in the absence of apparent inhibition of protein degradation, is not sufficient enough to block viral replication. However, additional reduction of recycled free ubiquitin by DUB inhibition can further enhance the inhibitory effect of proteasome inhibitor.

### CVB3 RNA-dependent RNA polymerase 3D is ubiquitinated

**Figure 5:**
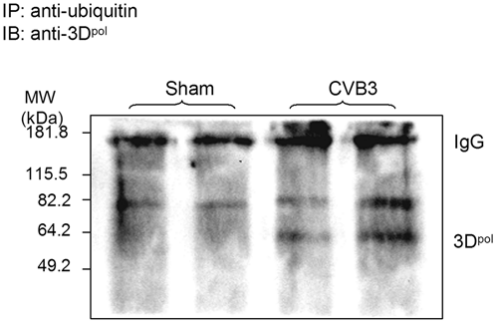
CVB3 RNA-dependent RNA polymerase 3D is ubiquitinated. HeLa cells were infected with CVB3 or sham-infected with PBS for 7 h, Cell lysates were collected and immuoprecipitated (IP) with a monoclonal anti-ubiquitin antibody. Protein-ubiquitin conjugates were detected by immunoblots (IB) using a polyclonal anti-3D^pol^ antibody. Immunoblot for antibody IgG was shown as loading controls. Similar results were observed in three independent experiments.

Some virus RNA-dependent RNA polymerases including the sindbis virus and the turnip yellow mosaic virus RNA polymerases have been demonstrated to be phosphorylated and ubiquitinated [31]. Although the role of ubiquitination of these RNA polymerases in the regulation of virus replication remains to be determined, such observation raises the interesting possibility that the ubiquitin/proteasome system may regulate CVB3 replication through ubiquitinating viral polymerase 3D, which is essential for initiating viral RNA replication. To examine whether coxsackieviral proteins are subjected to ubiquitination during viral infection, we performed immunoprecipitation with anti-ubiquitin antibody, followed by immunoblots using antibodies against 3D^pol^ and viral capsid protein VP1, respectively. As shown in Fig. 5, immunoreactive bands of around 60 kDa were detected in CVB3-infected cells. Non-modified 3D^pol^ has a molecular weight of about 53 kDa, thus this observation suggests that 3D^pol^ likely undergoes post-translational modification by monoubiquitination. No protein ubiquitination was observed for VP1 (data not shown). Our results implicate that the ubiquitination process of CVB3 viral proteins might be required for successful replication of the virus.

### Effects of CVB3 infection on protein expression of several key enzymes involved in the process of ubiquitination and deubiquitination

In trying to understand the mechanisms by which CVB3 manipulates the UPS, we examined the protein expression of several key enzymes involved in the process of protein ubiquitination and deubiquitination. We measured expression levels of ubiquitin-activating enzyme E1A/E1B, ubiquitin-conjugating enzyme Ubc H7, ubiquitin C-terminal hydrolase and two p53-related E3 ligases, human papillomavirus E6-associated protein and mouse double minute 2 homolog. However, no apparent changes were observed during the time-course of CVB3 infection (data not shown). These results indicate that the manipulation of the UPS by CVB3 is unlikely regulated by the above-examined ubiquitin-related key enzymes or molecules. Future studies will determine whether CVB3 infection targets on specific ubiquitin ligases or deubiquitinating enzymes.

## Discussion

In the present study, we have provided further evidence that CVB3 manipulates the UPS for its infection. CVB3 infection results in increased protein polyubiquitination and a subsequent decrease in free ubiquitin levels. Knockdown of ubiquitin and ubiquitin-mediated protein modification and/or degradation by siRNA markedly reduces CVB3 replication in HeLa cells, further supporting the essential roles of the UPS in the replication of CVB3.

It is increasingly apparent that viruses can evolve various strategies to utilize the host UPS for their own benefits. The UPS has been suggested to play a critical role in the different steps of viral lifecycle, including viral entry, viral replication, maturation, viral progeny release, and latent virus reactivation [32]–[34]. The mechanisms that the UPS regulates viral infection involve degrading intracellular proteins or excessive viral proteins that are against efficient viral replication and modulating viral protein function through ubiquitin-mediated modification or by directly encoding ubiquitin-related enzymes [35].

The finding in this study that CVB3 infection stimulates protein ubiquitination without inhibition of the core 20S proteasome activity highlights the possibility that CVB3 manipulates the UPS to destabilize or modulate the host and viral proteins. Polyubiquitination and degradation of host antiviral proteins has been suggested to be a mechanism of HIV-1 replication [36] . We have previously identified several proteins, such as cyclin D1, p53 and β-catenin, which are downregulated through the UPS after CVB3 infection [7], [9]. Destabilization of these short-lived host proteins is likely required for CVB3 viral RNA and protein synthesis in its lifecycle. Moreover, it is speculated that nonstructural viral proteins of CVB3 could also be potential targets of the UPS for degradation. Previous studies on picornavirus have shown that several viral proteins, such as encephalomyocarditis virus (EMCV) 3C protease and hepatitis A virus (HAV) 3C protease, are ubiquitinated and present in low concentrations in infected cells [37]–[39]. Several E3 ubiquitin ligases, such as human E3α ubiquitin ligase, have been shown to catalyze the ubiquitination of these viral proteins [38], [39]. Although the exact role of ubiquitination and subsequent degradation of nonstructural viral proteins of EMCV and HAV in infected cells remains elusive, such rapid turnover may be required for efficient viral RNA replication, viral protein synthesis and virus maturation.

As alluded to earlier, DUBs are a large family of cysteine protease responsible for the removal of ubiquitin from substrate proteins [40]. It is estimated that the human genome encodes more than 100 DUBs. Although UCHL1 is identified as an important DUB, inhibition of UCHL1 alone has been shown to only partially block the activities of DUBs [41]. Thus, the finding in this study that UCHL1/L3 inhibition is not as efficient in blocking viral replication as general inhibition of proteasome function or knockdown of ubiquitin is likely attributed to incomplete inhibition of DUBs by UCHL1/L3 inhibitors.

In addition to protein degradation, ubiquitin-modification has been suggested to be involved in the regulation of protein function. It was reported that monoubiquitination of the Gag protein of retroviruses is required for virus budding [22], [24], [25]. Depletion of free ubiquitin by proteasome inhibitors prevents Gag ubiquitination, subsequently blocks virus progeny release/budding. In addition, ubiquitination of human immunodeficiency virus type 1 Tat protein and human T-cell leukemia virus type 1 Tax protein has been shown to modulate their transactivation activities [20], [23]. We speculate that monoubiquitination is also an important machinery for post-translational modification and activation of CVB3 viral proteins. In the current study, we have shown that CVB3 RNA-dependent RNA polymerase 3D is post-translationally modified by ubiquitination, suggesting a critical role of protein ubiquitination in the regulation of viral protein functions.

**Figure 6:**
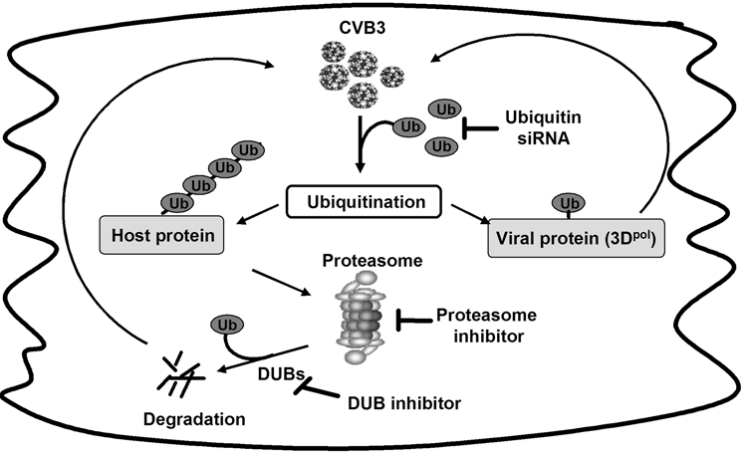
A proposed model for UPS regulation of CVB3 replication (See text). Abbreviation: CVB3, coxsackievirus B3; Ub, ubiquitin; DUBs, deubiquitinating enzymes; siRNA, small-interfering RNA; 3D^pol^, coxsackievirus RNA-dependent RNA polymerase 3D.

Based on the results in the manuscript, in combination of our previous findings that CVB3 infection promotes host protein degradation, including cyclin D1, p53 and β-catenin, a model system on the role of the UPS in CVB3 replication is proposed in Fig. 6. Coxsackievirus infection facilitates host protein polyubiquitination, which subsequently increases intracellular protein degradation by the proteasome and/or viral protein modification, such as 3D^pol^, by monoubiquitination. Degradation of host antiviral proteins provides a favorable environment for virus to achieve successful replication. Knockdown of ubiquitin decreases host protein degradation and viral protein ubiquitination. Proteasome inhibition blocks host protein degradation and viral protein ubiquitination by reducing recycled ubiquitin. DUB inhibitors further decreases the viral replication when used together with proteasome inhibitors through the additional reduction of recycled free ubiquitin.

In conclusion, we have demonstrated for the first time that CVB3 infection results in increased protein ubiquitination and consequent decreases in free ubiquitin levels. We further demonstrate that protein ubiquitination is required for the completion of viral lifecycle, likely through ubiquitin modification of viral polymerase.

## Materials and Methods

### Cell culture, virus, and materials

HeLa cells (American Type Culture Collection) were grown and maintained in complete medium [Dulbecco's modified Eagle's media (DMEM) supplemented with 10% heat-inactivated newborn calf serum (NCS) (Invitrogen)]. CVB3 (Kandolf strain) was propagated in HeLa cells and stored at −80°C. Virus titer was routinely determined by a plaque assay prior to infection as described below.

The monoclonal anti-β-actin and anti-ubiquitin antibodies were purchased from Sigma-Aldrich. The monoclonal anti-VP1 antibody was obtained from DakoCytomation. The ubiquitin siRNA, scramble control siRNA, and horseradish peroxidase-conjugated secondary antibodies were obtained from Santa Cruz Biotechnology. The proteasome inhibitors, MG132 and lactacystin, the UCH L1 inhibitor (LDN-57444) and the UCH L3 inhibitor (4,5,6,7-Tetrachloroindan-1,3-dione), and the polyclonal anti-ubiquitin antibody were obtained from Calbiochem. The polyclonal anti-3D^pol^ antibody was a generous gift from Dr. Karin Klingel (University Hospital Tuebingen, Germany).

### Virus infection

HeLa cells were grown in complete medium to 70–80% confluence, and then infected at a multiplicity of infection (MOI) of 10 with CVB3 or sham-infected with phosphate-buffered saline (PBS) for 1 h in serum-free DMEM. Cells were then washed with PBS and cultured in serum-free medium. For inhibition experiments, HeLa cells were infected with CVB3 for 1 h, washed with PBS, and then incubated with DMEM containing various concentrations of inhibitors.

### Immunoprecipitation and immunoblot analysis

Cell lysates were prepared using lysis buffer (50 mM pyrophosphate, 50 mM NaF, 50 mM NaCl, 5 mM EDTA, 5 mM EGTA, 100 µM Na_3_VO_4_, 10 mM HEPES (pH 7.4), 0.1% Triton X-100, and the protease inhibitor cocktail) as described previously [2]. For immunoblot analysis, equal amounts of protein were subjected to sodium dodecyl sulfate-polyacrylamide gel electrophoresis (SDS-PAGE) and then transferred to nitrocellulose membranes (GE Healthcare). Membranes were blocked for 1 h with nonfat dry milk solution (5% in PBS) containing 0.1% Tween 20. Blots were then incubated for 1 h with the primary antibody followed by incubation for 1 h with the secondary antibody. Immunoreactive bands were visualized by enhanced chemiluminescence (GE Healthcare). When protein ubiquitination was examined, membrane was heat-activated by autoclaving at 121°C for 35 min prior to blocking with nonfat dry milk solution to enhance antigenic site recognition.

For immunoprecipitation, cells were lysed using the above-described lysis buffer with freshly added 20 mM iodoacetamide. A total of 500 µg of cell lysates were incubated with a monoclonal anti-ubiquitin antibody (1∶100) at 4°C overnight, followed by 2 h incubation with protein G-agorose beads (Amersham). Immunocomplexes were washed five times with the lysis buffer containing 20 mM iodoacetamide, and then boiled for 5 min in the 2× non-reducing sample buffer which lacks both β-mercaptoethanol and DTT, but with addition of 20 mM iodoacetamide. After centrifugation, the precipitated proteins were separated by SDS-PAGE. Ubiquitin conjugates were analyzed by immunoblot using polyclonal anti-3D^pol^ antibody.

### Viral RNA *in situ* hybridization

HeLa cells were grown and maintained on two-chamber culture slides (Becton Dickinson Labware). Subconfluent cells were infected with either PBS or CVB3 (MOI = 10). Following 1 h of incubation at 37°C, cells were washed with PBS and replenished with complete medium in the absence and presence of MG132. HeLa cells were incubated for an additional 6 h. The culture slides were then washed gently with PBS, fixed with formalin buffer for 15 min, and then air-dried at room temperature. Culture slides were then subjected to *in situ* hybridization assays to detect the sense-strand of CVB3 genomic RNA as previously described [26].

### Plaque assay

CVB3 titer in cell supernatant was determined on monolayers of HeLa cells by an agar overlay plaque assay in triplicate as described previously [2]. Briefly, samples were serially diluted and overlaid on monolayer of HeLa cells. After 1 h incubation, medium was replaced with complete medium containing 0.75% agar. Cells were incubated for 72 h, then fixed with Carnoy's fixative (75% ethanol-25% acetic acid), and stained with 1% crystal violet. Plaques were counted and viral titer was calculated as plaque forming unit (PFU) per milliliter.

### Cell Viability Assay

MTS (3, 4-(5-dimethylthiazol-2-yl)-5-(3-carboxymethoxy phenyl)-2-(4-sulfophenyl)-2H-tetrazolium salt, Promega) assay was performed to determine cell viability as previously described [7]. Briefly, cells were incubated with MTS solution for 2 h prior to collection. Absorbance was measured at a wave length of 490 nm using an ELISA reader.

### Ubiquitin siRNA transfection

HeLa cells were grown to 50% confluency and then transiently transfected with ubiquitin-specific siRNA (200 nM) using oligofectamine according to the manufacturer's suggestion (Invitrogen). A scramble siRNA (200 nM) was used as a control. The silencing efficiency was detected by immunoblot analyses using the anti-ubiquitin antibody. After 24 h of transfection, cells were infected with CVB3 as indicated.

### Statistical analysis

Statistical analysis was performed using the paired *Student's t* test. A *p* value of less than or equal to 0.05 was considered statistically significant.

## References

[1] Opavsky MA, Martino T, Rabinovitch M, Penninger J, Richardson C (2002). Enhanced ERK-1/2 activation in mice susceptible to coxsackievirus-induced myocarditis.. J Clin Invest.

[2] Luo H, Yanagawa B, Zhang J, Luo Z, Zhang M (2002). Coxsackievirus B3 replication is reduced by inhibition of the extracellular signal-regulated kinase (ERK) signaling pathway.. J Virol.

[3] Cunningham KA, Chapman NM, Carson SD (2003). Caspase-3 activation and ERK phosphorylation during CVB3 infection of cells: influence of the coxsackievirus and adenovirus receptor and engineered variants.. Virus Res.

[4] Liu P, Aitken K, Kong YY, Opavsky MA, Martino T (2000). The tyrosine kinase p56lck is essential in coxsackievirus B3-mediated heart disease.. Nat Med.

[5] Fuse K, Chan G, Liu Y, Gudgeon P, Husain M (2005). Myeloid differentiation factor-88 plays a crucial role in the pathogenesis of Coxsackievirus B3-induced myocarditis and influences type I interferon production.. Circulation.

[6] Esfandiarei M, Luo H, Yanagawa B, Suarez A, Dabiri D (2004). Protein kinase B/Akt regulates coxsackievirus B3 replication through a mechanism which is not caspase dependent.. J Virol.

[7] Yuan J, Zhang J, Wong BW, Si X, Wong J (2005). Inhibition of glycogen synthase kinase 3beta suppresses coxsackievirus-induced cytopathic effect and apoptosis via stabilization of beta-catenin.. Cell Death Differ.

[8] Si X, McManus BM, Zhang J, Yuan J, Cheung C (2005). Pyrrolidine dithiocarbamate reduces coxsackievirus B3 replication through inhibition of the ubiquitin-proteasome pathway.. J Virol.

[9] Luo H, Zhang J, Dastvan F, Yanagawa B, Reidy MA (2003). Ubiquitin-dependent proteolysis of cyclin D1 is associated with coxsackievirus-induced cell growth arrest.. J Virol.

[10] Wolf DH, Hilt W (2004). The proteasome: a proteolytic nanomachine of cell regulation and waste disposal.. Biochim Biophys Acta.

[11] Schwartz AL, Ciechanover A (1999). The ubiquitin-proteasome pathway and pathogenesis of human diseases.. Annu Rev Med.

[12] Amerik AY, Hochstrasser M (2004). Mechanism and function of deubiquitinating enzymes.. Biochim Biophys Acta.

[13] Nijman SM, Luna-Vargas MP, Velds A, Brummelkamp TR, Dirac AM (2005). A genomic and functional inventory of deubiquitinating enzymes.. Cell.

[14] Glotzer M, Murray AW, Kirschner MW (1991). Cyclin is degraded by the ubiquitin pathway.. Nature.

[15] Pagano M, Tam SW, Theodoras AM, Beer-Romero P, Del Sal G (1995). Role of the ubiquitin-proteasome pathway in regulating abundance of the cyclin-dependent kinase inhibitor p27.. Science.

[16] Scheffner M, Werness BA, Huibregtse JM, Levine AJ, Howley PM (1990). The E6 oncoprotein encoded by human papillomavirus types 16 and 18 promotes the degradation of p53.. Cell.

[17] Palombella VJ, Rando OJ, Goldberg AL, Maniatis T (1994). The ubiquitin-proteasome pathway is required for processing the NF-kappa B1 precursor protein and the activation of NF-kappa B.. Cell.

[18] Roos-Mattjus P, Sistonen L (2004). The ubiquitin-proteasome pathway.. Ann Med.

[19] Hicke L (2001). Protein regulation by monoubiquitin.. Nat Rev Mol Cell Biol.

[20] Bres V, Kiernan RE, Linares LK, Chable-Bessia C, Plechakova O (2003). A non-proteolytic role for ubiquitin in Tat-mediated transactivation of the HIV-1 promoter.. Nat Cell Biol.

[21] Ott DE, Coren LV, Chertova EN, Gagliardi TD, Schubert U (2000). Ubiquitination of HIV-1 and MuLV Gag.. Virology.

[22] Patnaik A, Chau V, Wills JW (2000). Ubiquitin is part of the retrovirus budding machinery.. Proc Natl Acad Sci U S A.

[23] Peloponese JM, Iha H, Yedavalli VR, Miyazato A, Li Y (2004). Ubiquitination of human T-cell leukemia virus type 1 tax modulates its activity.. J Virol.

[24] Schubert U, Ott DE, Chertova EN, Welker R, Tessmer U (2000). Proteasome inhibition interferes with gag polyprotein processing, release, and maturation of HIV-1 and HIV-2.. Proc Natl Acad Sci U S A.

[25] Strack B, Calistri A, Accola MA, Palu G, Gottlinger HG (2000). A role for ubiquitin ligase recruitment in retrovirus release.. Proc Natl Acad Sci U S A.

[26] Luo H, Zhang J, Cheung C, Suarez A, McManus BM (2003). Proteasome inhibition reduces coxsackievirus B3 replication in murine cardiomyocytes.. Am J Pathol.

[27] Mimnaugh EG, Chen HY, Davie JR, Celis JE, Neckers L (1997). Rapid deubiquitination of nucleosomal histones in human tumor cells caused by proteasome inhibitors and stress response inducers: effects on replication, transcription, translation, and the cellular stress response.. Biochemistry.

[28] Jentsch S, Seufert W, Hauser HP (1991). Genetic analysis of the ubiquitin system.. Biochim Biophys Acta.

[29] Kirschner LS, Stratakis CA (2000). Structure of the human ubiquitin fusion gene Uba80 (RPS27a) and one of its pseudogenes.. Biochem Biophys Res Commun.

[30] Wilkinson KD (1997). Regulation of ubiquitin-dependent processes by deubiquitinating enzymes.. Faseb J.

[31] de Groot RJ, Rumenapf T, Kuhn RJ, Strauss EG, Strauss JH (1991). Sindbis virus RNA polymerase is degraded by the N-end rule pathway.. Proc Natl Acad Sci U S A.

[32] Morita E, Sundquist WI (2004). Retrovirus budding.. Annu Rev Cell Dev Biol.

[33] Shackelford J, Pagano JS (2004). Tumor viruses and cell signaling pathways: deubiquitination versus ubiquitination.. Mol Cell Biol.

[34] Furman MH, Ploegh HL (2002). Lessons from viral manipulation of protein disposal pathways.. J Clin Invest.

[35] Fan Z, Zhuo Y, Tan X, Zhou Z, Yuan J (2006). SARS-CoV nucleocapsid protein binds to hUbc9, a ubiquitin conjugating enzyme of the sumoylation system.. J Med Virol.

[36] Yu X, Yu Y, Liu B, Luo K, Kong W (2003). Induction of APOBEC3G ubiquitination and degradation by an HIV-1 Vif-Cul5-SCF complex.. Science.

[37] Losick VP, Schlax PE, Emmons RA, Lawson TG (2003). Signals in hepatitis A virus P3 region proteins recognized by the ubiquitin-mediated proteolytic system.. Virology.

[38] Lawson TG, Gronros DL, Evans PE, Bastien MC, Michalewich KM (1999). Identification and characterization of a protein destruction signal in the encephalomyocarditis virus 3C protease.. J Biol Chem.

[39] Lawson TG, Sweep ME, Schlax PE, Bohnsack RN, Haas AL (2001). Kinetic analysis of the conjugation of ubiquitin to picornavirus 3C proteases catalyzed by the mammalian ubiquitin-protein ligase E3alpha.. J Biol Chem.

[40] Love KR, Catic A, Schlieker C, Ploegh HL (2007). Mechanisms, biology and inhibitors of deubiquitinating enzymes.. Nat Chem Biol.

[41] Gong B, Cao Z, Zheng P, Vitolo OV, Liu S (2006). Ubiquitin hydrolase Uch-L1 rescues beta-amyloid-induced decreases in synaptic function and contextual memory.. Cell.

